# Chemical abscess post vein of Galen aneurysmal malformation embolisation with ethylene vinyl alcohol copolymer

**DOI:** 10.4102/sajr.v28i1.2841

**Published:** 2024-04-30

**Authors:** Uday Bhanu Kovilapu, Rahul Dudhal, Saurabh Maheshwari, Peeyush Dhagat, Umesh K. Mishra

**Affiliations:** 1 Department of Radiodiagnosis, Armed Forces Medical College, Pune, India

**Keywords:** vein of Galen, endovascular embolisation, ethylene vinyl alcohol copolymer (onyx), chemical abscess, onyx granuloma, brain abscess, VGAM, vein of Galen malformation

## Abstract

Vein of Galen aneurysmal malformation (VGAM) is a rare congenital malformation characterised by arteriovenous fistulas between primitive choroidal arteries and the median prosencephalic vein, the embryonic precursor to the vein of Galen. Endovascular techniques have changed the management of these patients with improved prognosis. An eight-month-old with VGAM managed by endovascular embolisation using ethylene vinyl alcohol copolymer (EVOH) developed a chemical abscess - a rare complication. It was managed conservatively and showed promising clinical outcome.

**Contribution:**

Chemical abscesses following EVOH embolisation are scarce – with imaging differentials, which include brain abscess and onyx granuloma. Knowledge and successful identification of this entity are essential as its management as prognoses differ. Chemical abscess is managed conservatively and has a good prognosis.

## Introduction

Vein of Galen aneurysmal malformations (VGAM) are rare anomalies of the intracranial vasculature, constituting 37.0% of paediatric vascular malformations.^1^ They are characterised by arteriovenous fistulas between primitive choroidal arteries and the median prosencephalic vein, the embryonic precursor to the vein of Galen (VOG). The fistula prevents the regression of the median prosencephalic vein and the development of the vein of Galen. It is associated with poor clinical outcomes, with a mortality of 76.7% if left untreated.^2^ With the advent of newer techniques and management strategies, the overall survival with endovascular treatment and technical success is approximately 80.0%.^3,4^ This report describes a rare complication of onyx embolisation, a chemical abscess, which was managed conservatively and has favourable long-term results.

## Case report

An 8-month-old child presented with progressively increasing head size since birth, global developmental delay in achieving age-appropriate milestones (the child’s milestones were equivalent to 3 months as per accepted developmental milestones), and cranial bruits. The occipito-frontal circumference was 54 cm (standard for age is 43 cm–45 cm).

**FIGURE 1:**
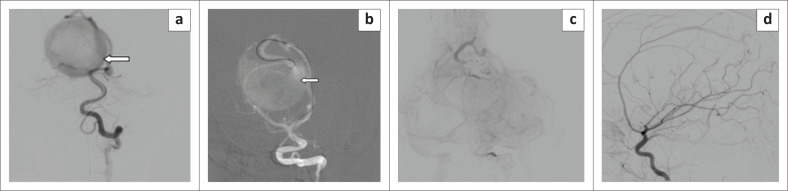
Digital subtraction angiography images: (a) Left vertebral artery run showing the posteromedial choroidal branch (arrow) of left posterior cerebral artery (PCA) feeding the vein of Galen aneurysmal malformation sac. (b) Endovascular selective cannulation of the posteromedial choroidal artery branches of the left PCA. (c) Onyx cast following the slow injection of concentrated onyx. (d) Immediate post-procedure angiogram showed marked slowing of flow within the dilated median vein.

Magnetic resonance imaging of the brain revealed a well-circumscribed markedly T2 hypo-intense midline lesion dorsal to the third ventricle, causing mass effect on the aqueduct with resultant dilatation of the lateral and third ventricles and a normal-sized fourth ventricle suggestive of obstructive hydrocephalus. Cerebral digital subtraction angiography confirmed a mural type of VGAM (Lasjaunias classification) with feeders from both posterior cerebral arteries and the left anterior cerebral artery (Figure 1).

### Management and outcome

**FIGURE 2:**
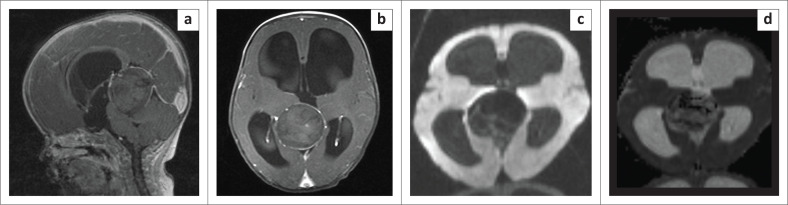
Follow-up MRI on day 3 post-procedure: (a) T1-fat-saturated post-contrast mid-sagittal, (b) T1-fat-saturated post-contrast axial, (c) Diffusion-weighted imaging axial at b 1000 s/mm^2^ and (d) Apparent diffusion coefficient axial at the same level as (c); showing the vein of Galen aneurysmal malformation thrombosed sac with hydrocephalus, mild peripheral rim enhancement and absence of restricted diffusion.

The child proceeded to endovascular management. Right femoral arterial access was acquired with a 5 French(Fr) short sheath, and a 5 Fr guiding catheter was placed through the short sheath and negotiated into the right vertebral artery (V4 Segment). Following this, super selective cannulation of the posteromedial choroidal branches of the right posterior cerebral artery (PCA) was accomplished, and 2 mL of onyx 18 was injected on the arterial side with reflux into the large venous sac, resulting in its occlusion. Complete avascularity of the sac was achieved after occluding the VOG sac. Post embolisation MRI on day three revealed a minimal reduction in the size of the VOG sac, with minimal enhancement of the sac wall and no internal restricted diffusion, as shown in Figure 2.

**FIGURE 3:**
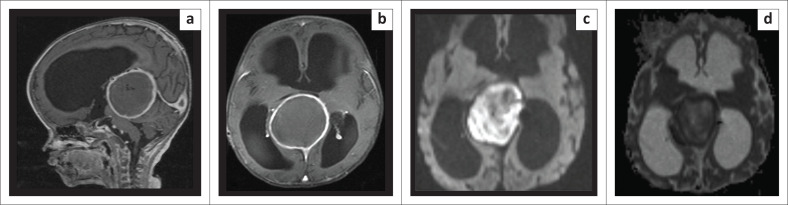
Follow-up MRI at 1-month post-procedure demonstrating the chemical abscess: (a) T1FS post-contrast mid-sagittal, (b) T1-fat-saturated post-contrast axial, (c) Diffusion-weighted imaging axial at b 1000 s/mm^2^ and (d) Apparent diffusion coefficient axial at the same level as 3c; showing a mild reduction in the size of the aneurysmal sac, persistent hydrocephalus, thick peripheral mural enhancement and marked restricted diffusion.

The child was followed up at regular intervals for 2 years. There was a gradual improvement in achieving developmental milestones. Follow-up MRI after 1 month revealed a residual aneurysmal sac showing wall enhancement, restricted diffusion, and persistent hydrocephalus, as depicted in Figure 3.

**FIGURE 4:**
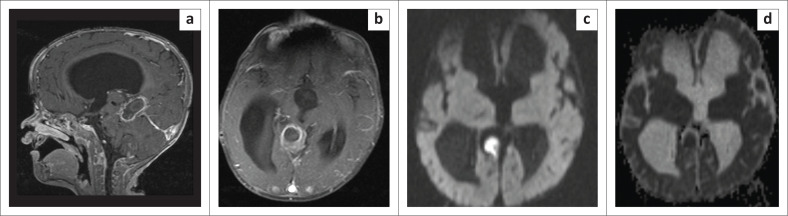
Follow-up MRI at 6 months post-procedure showed a resolving chemical abscess: (a) T1-fat-saturated post-contrast mid-sagittal, (b) T1-fat-saturated post-contrast axial, (c) Diffusion-weighted imaging axial at b 1000 s/mm^2^ and (d) Apparent diffusion coefficient axial at same level as 4c showing further reduction in the size of the remnant sac and hydrocephalus, persistent peripheral rim enhancement and restricted diffusion.

A differential diagnosis of brain abscess was considered; however, in view of the gradual improvement in cognitive function, lack of clinical deterioration, and lack of oedema in the surrounding brain parenchyma on MRI, a diagnosis of chemical abscess was made. The child was managed conservatively and followed up. There was further clinical improvement at 6 months post embolisation {developmental milestones equivalent to 10 months of age as per the Centres for Disease Control and Prevention (CDC) criteria}. On MRI there was a reduction in the size of the residual sac and hydrocephalus, albeit with continued restricted diffusion and peripheral mural enhancement, as shown in Figure 4.

After 2 years, the child had achieved milestones commensurate with age. The head circumference was stabilised on examination, and no neurological deficits were present. At this stage, MRI revealed near-total regression of the sac with no residual wall enhancement.

## Discussion

Vein of Galen aneurysmal malformation is associated with poor clinical outcomes, with a mortality rate of 76.7% if left untreated.^2^ Various treatment modalities include endovascular repair, microsurgery (craniotomy and vascular clip occlusion), and gamma knife surgery. Microsurgery and gamma knife surgery have been reported to have very high mortality and morbidity.^5^ Given this, endovascular techniques have emerged as the standard of care for treating VGAM because of the low complication rate and favourable outcome.^3,4^

The options for approaching the embolisation include transarterial, transvenous and transocular, with higher complications reported in the latter two approaches.^6,7^ Previously, glue (n-butyl cyanoacrylate) was the standard embolic liquid agent used; however, its characteristics limit precise control of the material during injections. Ethylene vinyl alcohol copolymer (EVOH), dissolved in dimethyl sulfoxide (DMSO) and suspended micronized tantalum powder for visualisation under fluoroscopy, allows a more controlled and more precise embolisation as it is less adhesive and polymerises slowly. It has recently substituted glue for embolising various arteriovenous malformations,^8,9^ and was also used in the patient presented. Technical complications are more common in neonates than infants. The complications include near vessel occlusion, haemorrhage and catheter retention.

In the current case, the infant had a mural type of VGAM. Post-embolisation follow-up MRI after 1 month revealed a residual aneurysmal sac showing wall enhancement, restricted diffusion, and persistent hydrocephalus. Brain abscesses, and onyx granuloma formation have been described in case reports as rare complications after endovascular embolisation of brain arteriovenous malformations. They should be considered in the differential diagnosis as their management is different. Brain abscess requires drainage and specific antibiotic therapy. Onyx granulomas are usually self-limiting and require treatment only when progressive and symptomatic.^10^ Diffusion-weighted imaging has been recommended to differentiate between abscess and granuloma, with abscess showing restricted diffusion.^11^

The second and third follow-up MRI indicated thick mural enhancement and marked restricted diffusion. However, a benign aetiology of chemical abscess was considered as there was progressive clinical improvement, regaining of developmental milestones, no further decline in neurological development, and no oedema of the surrounding brain parenchyma on T2WI/FLAIR images. Subsequent follow-up 6 months post embolisation showed even further clinical improvement. Magnetic resonance imaging showed a reduction in the size of the residual sac and hydrocephalus, albeit with continued restricted diffusion and peripheral mural enhancement, as shown in Figure 4. The continuing clinical improvement and reduction in size confirmed the diagnosis. Further MRI after 2 years revealed near-total regression in the sac with no significant wall enhancement.

A possible cause of the persisting enhancement could be the inflammatory response in the walls of the embolised vessel because of the onyx material. Microscopically, multinucleated giant cells have been demonstrated in onyx granuloma.^11^ A rare complication of intravascular onyx injection causing the formation of the cerebral abscess has been reported.^12^ However, an infective aetiology was not considered in the case presented as there was no clinical deterioration or new neurological deficit. The diagnosis of chemical abscess was made based on the clinical and radiological findings, which consolidated and reduced in size over 2 years.

## Conclusion

The introduction of endovascular treatment has revolutionised the management of VGAM. Although chemical abscess formation after embolisation is exceedingly rare, it is a benign condition that should be differentiated from an infectious abscess. Interventional neuro-radiologists and paediatricians should be vigilant about this complication to avoid unnecessary intervention. However, patients will require regular follow-up to assess the course and regression of the chemical abscess.
